# Genome analysis of SARS-CoV-2 isolates from a population reveals the rapid selective sweep of a haplotype carrying many pre-existing and new mutations

**DOI:** 10.1186/s12985-023-02139-3

**Published:** 2023-09-01

**Authors:** Maloyjo Joyraj Bhattacharjee, Anupam Bhattacharya, Bhaswati Kashyap, Manash Jyoti Taw, Wen-Hsiung Li, Ashis K. Mukherjee, Mojibur Rohman Khan

**Affiliations:** 1 Division of Life Science, Institute of Advanced Study in Science and Technology, Paschim Boragaon, Guwahati, Assam 781035 India; 2 Department of Microbiology, Gauhati Medical College and Hospital, Guwahati, Assam 781032 India; 3 Biodiversity Research Center, Academia Sinica, 11529 Taipei, Taiwan; 4 Department of Ecology and Evolution, University of Chicago, Chicago, IL 60637 USA

**Keywords:** SARS-CoV2, Delta variant, Haplotypes, Selective sweep, ORF8

## Abstract

To understand the mechanism underlying the evolution of SARS-CoV-2 in a population, we sequenced 92 viral genomes from Assam, India. Analysis of these and database sequences revealed a complete selective sweep of a haplotype in Assam carrying 13 pre-existing variants, including a high leap in frequency of a variant on ORF8, which is involved in immune evasion. A comparative study between sequences of same lineage and similar time frames in and outside Assam showed that 10 of the 13 pre-existing variants had a frequency ranging from 96 to 99%, and the remaining 3 had a low frequency outside Assam. Using a phylogenetic approach to infer sequential occurrences of variants we found that the variant Phe120del on ORF8, which had a low frequency (1.75%) outside Assam, is at the base of the phylogenetic tree of variants and became totally fixed (100%) in Assam population. Based on this observation, we inferred that the variant on ORF8 had a selective advantage, so it carried the haplotype to reach the100% frequency. The haplotype also carried 32 pre-existing variants at a frequency from 1.00 to 80.00% outside Assam. Those of these variants that are more closely linked to the S-protein locus, which often carries advantageous mutations and is tightly linked to the ORF8 locus, retained higher frequencies, while the less tightly linked variants showed lower frequencies, likely due to recombination among co- circulating variants in Assam. The ratios of non-synonymous substitutions to synonymous substitutions suggested that some genes such as those coding for the S-protein and non-structural proteins underwent positive selection while others were subject to purifying selection during their evolution in Assam. Furthermore, we observed negative correlation of the Ct value of qRT-PCR of the patients with abundant ORF6 transcripts, suggesting that ORF6 can be used as a marker for estimating viral titer. In conclusion, our in-depth analysis of SARS-CoV-2 genomes in a regional population reveals the mechanism and dynamics of viral evolution.

**Supplementary Information:**

The online version contains supplementary material available at 10.1186/s12985-023-02139-3.

## Introduction

Severe Acute Respiratory Syndrome 2 (SARS-CoV-2) was first reported in Wuhan, China in late 2019, but has since evolved into several variants worldwide with variable transmissibility and pathogenicity [1]. According to World Health Organization (WHO), there are five variants of concern (VOC) globally: alpha (α), beta (β), gamma (γ), delta (δ), and omicron [2]. Reportedly, α, δ and the recently evolved omicron variant have shown increased transmissibility [3, 4].

Whole genome sequencing (wgs) of variants has greatly facilitated tracking the emergence of novel variants of SARS-CoV-2 [5]. The GISAID [6] and NCBI databases (https://www.ncbi.nlm.nih.gov/sars-cov-2) house around 13,758,184 and 6,416,687 wgs of SARS-CoV2, respectively (as on 3^rd^ November, 2022). The GISAID and Pangolin databases [7] assign a newly sequenced SARS-CoV-2 genome to a clade and a lineage. The α variant belongs to the GRY clade [6] that includes the lineages B.1.1.7 + Q* (Q* denotes all descendent lineages of Q) [7]. Similarly, the δ variant belongs to the clade GK that includes the lineages B.1.617.2 + AY*, and the recently evolved omicron variant belongs to the GRA clade that includes the lineages B.1.1.529 + BA*. The clade definitions (GRY, GK, GRA, etc.) in GISAID are based on the letters of marker mutations [7].

Although the global transmission of the virus has been tracked based on the designated VOCs mentioned above, it does not provide a deep insight into possible acquisition of selective advantage associated with temporal and geographical spread of the virus [8–10]. A few previous large-scale genomic studies have found upsurges of haplotypes of SARS-CoV-2 associated with a particular geographic region [11–13]. Nonetheless, some studies found that SARS-CoV-2 produces a highly mutant replication intermediate which expresses variant SARS-CoV-2 proteins in different populations [14–16]. The same replication intermediate also produces a high number of mutations and deletions across the genome, favoring quasispecies dynamics and also conferring immune evasion. It is apparent that the geographical and temporal spread of the virus has been driven by many unique changes in the genome that may provide selective advantages. A deep understanding of those genomic features will provide valuable information for effective pandemic responses in different regions rather than a less-effective universal response.

As part of global endeavour, we obtained whole genome sequences of 92 SARS-CoV-2 patients in Assam, India, for the surveillance of genome-wide mutations of SARS-CoV-2 from a regional perspective. We primarily focused on potentially advantageous mutations and hitch-hiking of linked mutations. We also studied the expression of SARS-CoV-2 genes to provide an insight into the effect of SARS-CoV-2 gene expression in the establishment of virus titre in human hosts. In addition, we studied genome-wide and gene-wise selection patterns in α, δ, and the omicron variants with a wider dataset. This study has significantly increased our understanding of regional transmission of SARS-CoV-2 variants. It also featured the role of expression of SARS-CoV-2 genes in the establishment of a viral load and the natural selection on those genes carrying SARS-CoV-2 variants. In addition, this study provided information on characteristic nucleotide and amino acid mutations that probably affected the dynamics of the evolution of SARS-CoV-2 in Assam, Northeast India.

## Results

### Phylogeny of derived and database sequences of SARS-CoV2

**Fig. 1:**
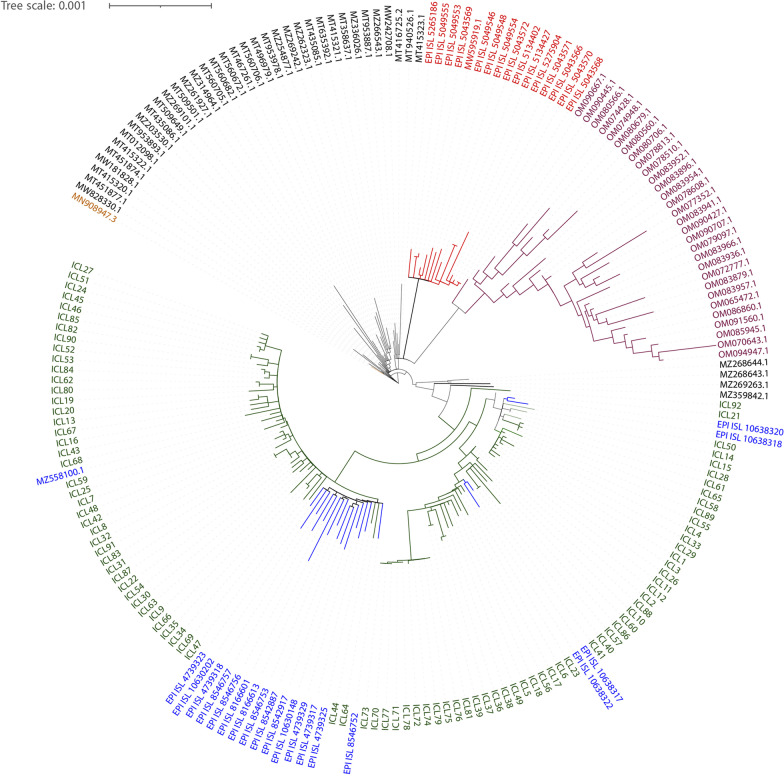
Maximum Likelihood phylogeny of new SARS-CoV-2 variants and database sequences. The reference genome from Wuhan (yellow) is found at the base. The genome sequences of the first wave (black) from India, α from UK (red), δ from different states of India (blue) collected from the NCBI and GISAID SARS-CoV-2 database, and omicron (purple) variants from USA and Bahrain are clustered in separate clades. All of the 92 sequences (green) from Assam derived in this study are denoted by ‘ICL’ followed by a number and they are clustered within the δ variant clade

The Phylogenetic Assignment of Named Global Outbreak Lineages (PANGOLIN) [7] database classified all our derived sequences to delta variant (Additional file 1: Table S1) and assigned them to the six lineages B.1.617.2 (27 patients), AY.33 (26), AY.16 (24), AY.4 (12), AY.34 (1), and AY.37 (1). This observation is represented by a phylogenetic tree (Fig. 1), where we used our derived genomic sequences and also representative sequences from the first wave (FW), α, δ, and omicron variants (Additional file 2: Table S2) because they are highly transmitted globally with characteristic pathogenesis, and represent variants of different pandemic timelines In this tree, the reference sequence (NC_045512) from Wuhan, China, is found at the base, and the database sequences from the FW are clustered closely with the Wuhan sequence. The sequences of the α variant that spread all over Europe, particularly in the UK, and the omicron sequences are clustered as distinct clades. Representative sequences of the δ variant are taken from the sequence pool submitted to the GISAID database from the states of India such as Maharashtra, Delhi, Uttar Pradesh, Gujrat, and Assam, which were highly infected by the δ variant (Additional file 2: Table S2). All the δ variant sequences formed a distinct clade and all the sequences derived from Assam were clustered within the δ variant clade. After the emergence of the Wuhan strain, the FW of SARS-CoV-2 persisted for around 7-8 months and several lineages evolved. From late 2020, the variants that posed an increased risk such as the α and the δ variants evolved. The sequences of the FW lineages B.1.1.5, B.1.153, B.1.617.1, and B.1.617.3 likely represented the basal clade from which the δ variant in India evolved. The clade of the δ variant sequences was divided into three sub-clades in Fig. 1, which is consistent with GISAID clades for the δ variant. The δ variant sequences derived in this study from Assam together with the sequences from other states segregated among the three sub-clades and most interestingly it also includes two sequences (ICL21 and ICL92) that are at the base of the δ variant clade. This suggests that some early infections occurred in or migrated to Assam.

### Mutations in the sequenced genomes

**Fig. 2:**
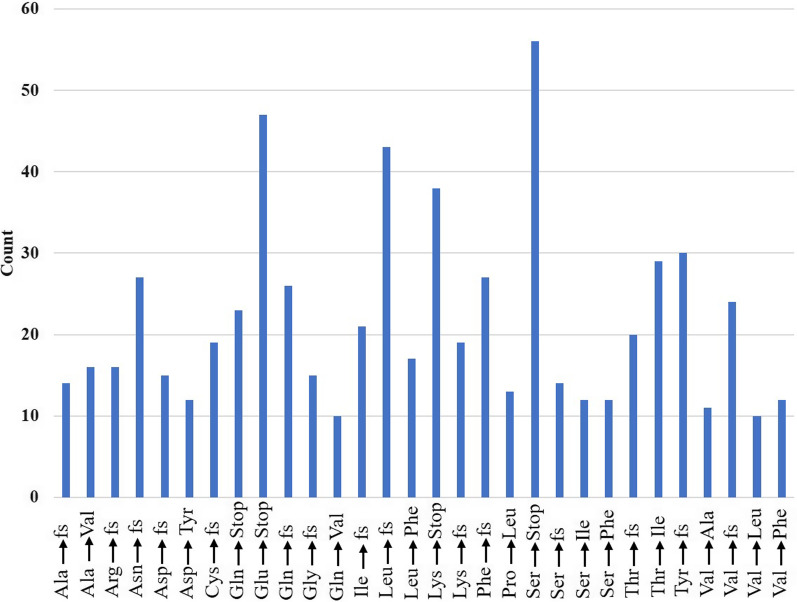
Genome-wide major amino acid variants observed in the 92 studied SARS-CoV-2 samples. The X-axis depicts major amino acid mutations while the Y-axis depicts the number of genomes (sequenced in this study) in which a mutation appeared. The dominant variants are either frame-shift (fs) mutations or changes to stop codons. The dataset is a collection of variants observed in the derived sequences against the reference sequence from Wuhan, China (accession NC_045512)

We next focused on nucleotide and amino acid changes in our studied genomes and compared them with –the GISAID Database of SARS-CoV-2 Variants (medbiotech-lab.ma). We aligned the reads of our samples and called variants using the GATK pipeline [17] with the Wuhan strain as the reference with the cut-variant quality score of 30 in Phred-scale. A genome-wide variation map is shown in Additional file 3: Fig. S1 and detailed in Additional file 4: Table S3, which shows a higher proportion of homozygous variants (59.02%) than that of heterozygous variants (40.98%). The relatively high heterozygosity implies that the virus sample extracted from a swab was a collection of viral particles whose sequences might differ across many regions in the genome. Altogether, 1071 nucleotide variants were observed in the studied samples (Additional file 5: Table S4), which may be categorized as (1) “conservative in-frame deletion”: 125; (2) “disruptive in-frame deletion”: 142; (3) “disruptive in-frame insertion”: 1; (4) “3’ end variant”: 116; (5) “frameshift”: 797; (6) “frameshift and start-codon loss”: 1; (7) “frameshift and stop-codon gain”: 30; (8) “mis-sense mutation”: 2808; (9) stop-codon gain: 8; (10) “stop-codon gain and disruptive in-frame deletion”: 1; (11) “synonymous mutation”: 695; and (12) “5’ end mutation”: 291. We found 977 nonsynonymous mutations, which may be categorized into 174 types (Additional file 5: Table S4), with 30 major patterns as shown in Fig. 2. Clearly, Ser-to-stop, Lys-to-stop, Glu-to- stop, Gln-to-stop, Leu-to-Phe, Leu-to-fs, Glu-to-fs, Thr-to-fs, and Val-to-fs types of amino acid changes occurred more frequently than the other types of mutations in the 92 genomes studied. The total variants found are shown in Additional file 6: Table S5.

### A selective sweep of a delta variant haplotype in the Assam population

**Fig. 3:**
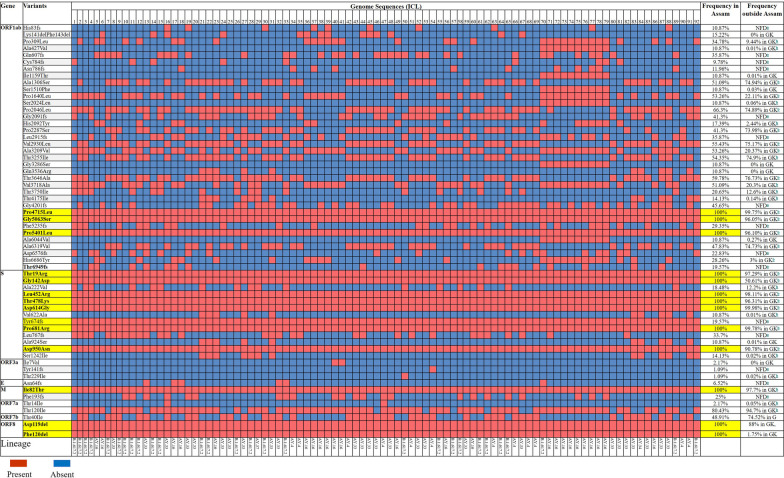
Major amino acid variants observed in the 92 sequenced SARS-CoV-2 genomes of Assam, India. The amino acid changes are represented, for example, by “Pro309Leu- ORF1ab”, which means ‘Pro’ changed to ‘Leu’ at position 309 of the ORF1ab polypeptide. In the second column, the high frequency variants are highlighted in yellow and the novel variants are indicated by *. The dark red and blue colours represent the presence and the absence of a particular variant in a particular genome denoted by ICL with a number in the top row, and each column represents a single sequence. ^a^NFD: “Not found in database”. ^b^GK refer to the sub-clade within the G clade of SARS-CoV2 in the GISAID database. The GK clade, according to the WHO nomenclature, refers to the δ variant. ‘fs’ and ‘del’ refer to frame-shift and deletion, respectively

**Fig. 4:**
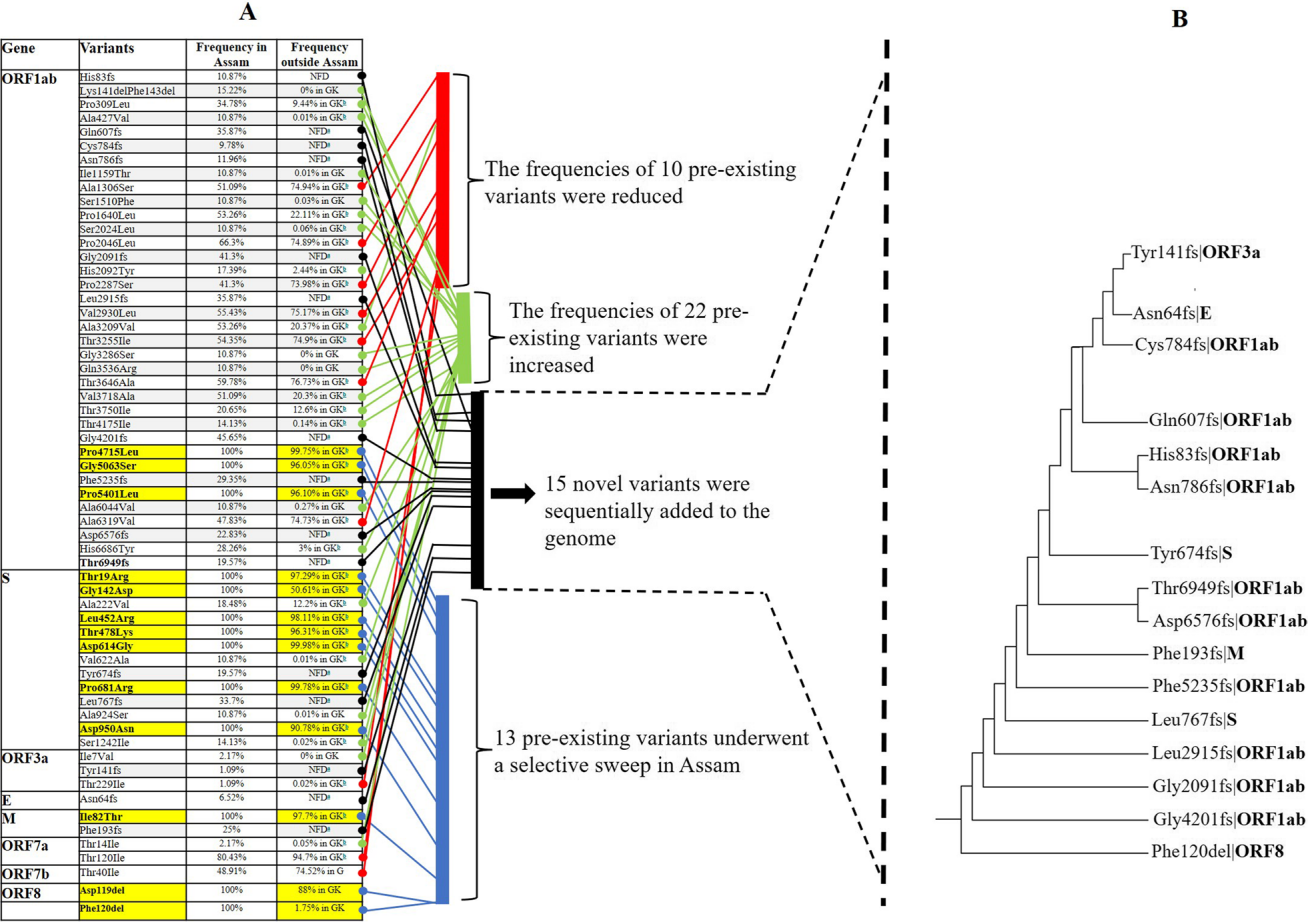
A proposed scheme for the evolution of the amino acid changes found in the 92 sequenced genomes in Assam. **A** A selective sweep and losses of variants. The amino acid changes are represented as in Fig. 3. A haplotype having a set of 13 underwent a selective sweep in Assam. **B** Sequential occurrences of frame-sift variants in the 92 SARS-CoV-2 genomes of Assam. Fifteen frame-shift variants (indicated by the black bar) arose sequentially and were added to the genomes at different time points. The tree is constructed by the Neighbour Joining (NJ) method using the presence and absence of variants in a particular genome as the distance measure. The basal variant represents the oldest variant that is fixed in the population of Assam. The frequencies of the variants and their occurrences in a particular gene are detailed in Fig. 3. * Frame-sift variant in a region of the genome not found in the GISAID databse

The majority of amino acid variants (mutations) and their frequency found in the SARS-CoV-2 lineages (B.1.617.2, AY.33, AY.16, AY.4, AY.34, and AY.37) detected in Assam and the corresponding frequencies of the variants to the same lineages from outside Assam over a similar time frame (March–July, 2021) are shown in Fig. 3 (also simplified in Additional file 7: Table S6 and Additional file 1: Table S1). Altogether, we found 60 major amino acid variants (Fig. 4a). Among them, 13 were high frequency (100%) variants in the 92 genomes from Assam, of which 9 variants appeared in the frequency range of 95–99%, 2 variants in the range of 88–90%, one variant with 50%, and one variant (Phe120del on ORF8, which means that Phe at 120 were deleted) with 1.75% appeared in the GK clade of GISAID outside Assam (Fig. 3). Moreover, besides the high frequency variants, there were 32 low/moderate frequency (1.09–80%) variants from Assam, of which 10 variants decreased in frequency and 22 variants slightly increased in frequency (indicated by a red and a green bar in Fig. 4a). This implies that a specific haplotype of the δ variant carrying 13 pre-existing amino acid variants (indicated by a blue bar in Fig. 4a) underwent a selective sweep in Assam, whereby three variants (Gly142Asp on S, Asp119del on ORF8, and Phe120del on ORF8) showed a significant leap in frequency, most notably Phe120del on ORF8. Thus, it was likely that the variant Phe120del on ORF8 singly or in conjugation with Gly142Asp on S and Asp119del on ORF8 had a selective advantage and carried the haplotype to fixation in Assam. Distinguishing between these two scenarios requires further study.

The above haplotype also carried 32 pre-existing variants (mutations) at a frequency from 1.00% to 80.00% (the variants indicated by the red bar in Fig. 4a). Among them, 10 variants were reduced in frequency and 22 variants slightly increased in frequency in Assam. These 32 variants included 4 pre- existing variants in S-protein, 2 variants in ORF3a, 2 variants in ORF7a, 1 variant in ORF7b, and 23 variants in ORF1ab. Except for a few cases, the variants which are more closely linked to the S-protein locus retained higher frequencies compared to the variants that are less tightly linked to the S-protein locus. Note that the S-protein locus and the ORF8 locus are neighbors, as shown in Fig. 4a. Thus, a variant tightly linked to the S-protein locus is also tightly linked to the ORF8 locus. Note further that, as noted above, the Asp119del and Phe120del on ORF8 was likely selectively advantageous. The spike (S) protein critically determines the entry of the virus into the human cell and 7 S-protein variants showed a frequency of 100% in Assam, of which 5 variants showed frequency in the range of 96–99%, and two variants Asp950Asn and Gly142Asp showed frequency of 90% and 50% respectively. The combination of these variants might be advantageous for the transmissibility of the virus in a population of Assam. However, it is possible that some of these variants did not have selective advantage but happened to be carried on the S-protein, that is, they were hitch-hiking variants. According to the principle of non-random assortment of variants, the variants associated with a trait may show strong linkage disequilibrium (LD) with other closely linked variants in a population [18]. The high-frequency variants in ORF1ab, M, and ORF8 closely linked to the S-protein locus (and thus also to the ORF8 locus) likely had a high LD with respect to the variants in S- protein. The variants which were only weakly linked to S-protein (and ORF8) variants had a weak LD and were therefore reduced in frequency (except a few cases) likely by recombination between co-circulating variants. Infection of the same person by two different variants of SARS-CoV-2 was reported previously from Assam [19], which likely provided a chance for recombination events. This is consistent with the previous reports that the evolution of SARS-CoV2 variants involved repeated episodes of recombination [20–22].

We also found many variants with a low/moderate frequency (variants indicated by the black bar in Fig. 4a), which are not found in the GISAID database and marked as NFD. Most of these NFD variants include frame- shift mutations. Figure 3 shows that a variant might appear with another variant on the same genomes. Here we assume that if the variant with the lower frequency appeared only on the sequences that carried the variant with a higher frequency, then the mutation (variant) occurred on a sequence that carried the variant with a higher frequency and thus was younger. Using this assumption, we inferred the sequential occurrences of 15 frame-shift variants in the 92 genomes of Assam (Fig. 4b) together with Phe120dellORF8, which has a very low frequency outside Assam. Figure 4b shows that Phe120dellORF8 at the base of the tree was totally fixed in the Assam SARS-CoV-2 population (bottom of Fig. 3) after which the 15 frame-shift variants sequentially evolved to spread across the population.

### Genomic epidemiology of the delta variant in rest of India

**Fig. 5:**
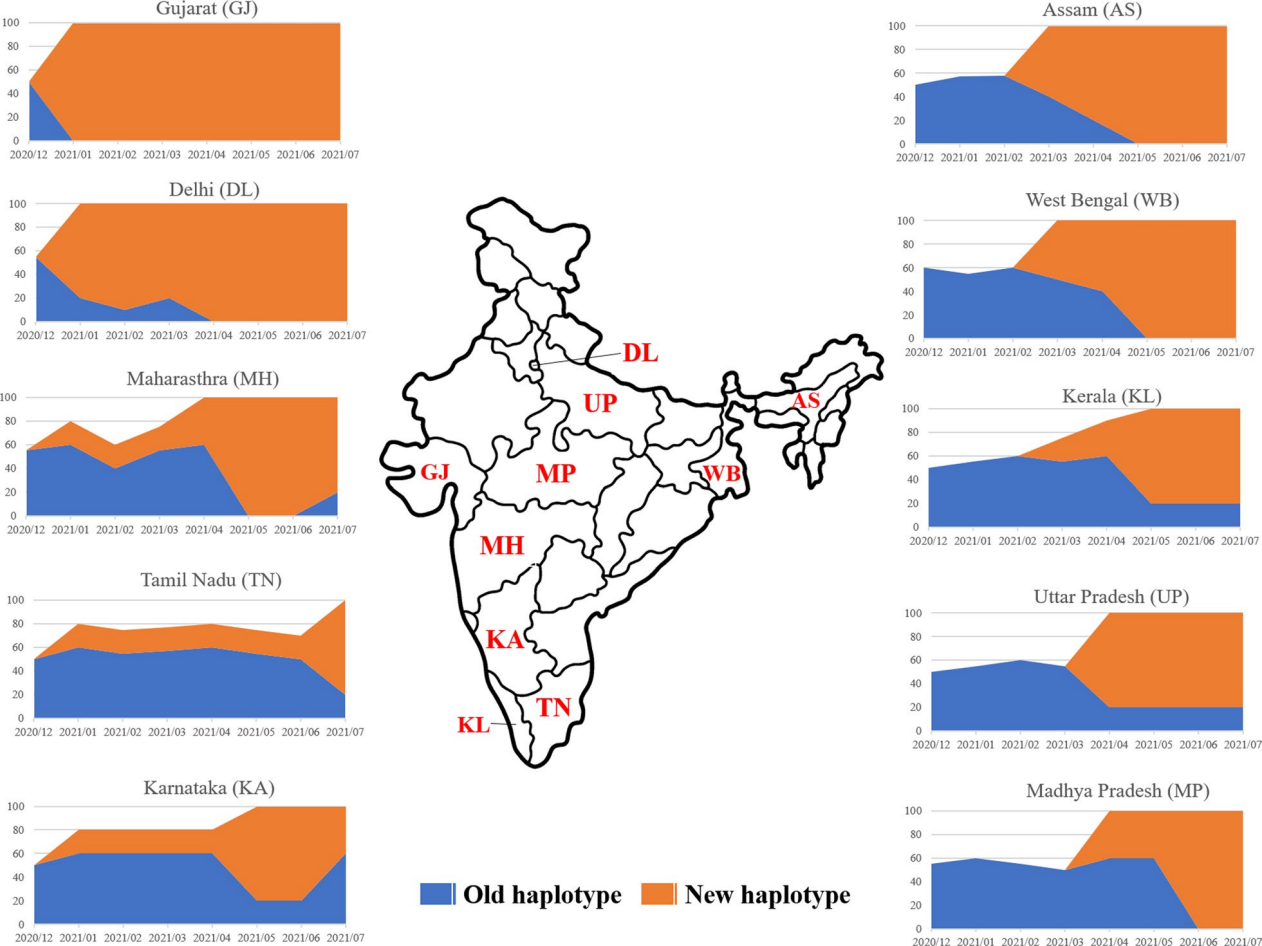
Map of SARS-CoV-2 delta variant haplotypes designated as ‘old haplotype’ and ‘new haplotype’ in this study. The frequency of the haplotypes in each of the Indian states are represented as stacked area chart to display their frequency (in percentage as shown in the vertical axis) over time (as shown in the horizontal axis)

The above findings showed that a haplotype of δ variant of SARS-CoV-2 underwent selective sweep with 13 amino acid variants. Since, out of the 13 variants, 11 are high frequency variants (88–99%), for the sake of convenience of explanation we designated the haplotype with 11 high-frequency variants as ‘old haplotype’ and the haplotype with the 11 high-frequency plus the low-frequency variants (Gly142Asp on S and Phe120del on ORF8) as the ‘new haplotype’. To understand the genomic epidemiology of δ variants in Assam and their connection to the rest of India, we checked the frequencies of the old haplotype and the new haplotype (if any) in other states of India. SARS-CoV-2 δ variant was first discovered in India in December, 2020 and it had strongly affected India from March to July, 2021. The Indian SARS-CoV-2 genomics (INSACOG) consortium and the GISAID database houses many δ variant sequences collected between December 2020 to July, 2021 from different high burden states of India. We accessed the sequences from ten high burden states such as West Bengal and Assam representing East and Northeast India, Kerala, Karnataka, and Tamil Nadu representing South India, Madhya Pradesh, Delhi, and Uttar Pradesh representing central India, and Maharashtra and Gujrat representing Southwest and West India, to check the frequency of the new (if present) and old haplotype. A map of the haplotype frequency among the Indian states is shown in Fig. 5. The figure revealed that, while the old haplotype was present among the δ variants in a frequency ranging from 50 to 60% sampled between December 2020 to February, 2021 when the infection rate was around 30,000/day, the haplotype eventually reduced over time July, 2021. Interestingly, the new haplotype showed a frequency in the range of 80–100% in high burden states of India sampled between March to July, 2021 when the infection rate was higher with the highest recorded daily cases in India > 4,14,000 (as on May 7, 2021) during the study period. Moreover, it is evident that the early cases of the new haplotype (January, 2021) appeared in higher frequency in Gujrat (100%) and Delhi (80%) and comparatively in lesser frequency in Maharashtra (20%), Karnataka 20%, and Tamil Nādu 20%. This clearly suggest that the new haplotype, has a clear selective advantage, so it spread very rapidly. Since, in Assam, the first case of δ variant was detected in around March, 2021, following a legislative assembly election with mass crowd gathering, the evolution of the new haplotype might have occurred outside Assam somewhere in Gujrat or Delhi, and migrated to Assam following crowd movement during the election period.

### Natural selection on genes of the SARS-CoV-2 samples

We studied the ratio of the non-synonymous substitutions (dN) to synonymous substitutions (dS), i.e., the dN/dS, among the samples of the first wave (FW) variant (January to June 2020), the α variant (November 2020 to February 2021), and the δ variant (February to June 2021) in Assam, and the omicron variant outside Assam (Table 1). The details of the sequences compared is given in Additional file 1: Table S1. We calculated the dS (number of synonymous substitutions per synonymous site) and dN (number of non-synonymous substitutions per non-synonymous site) using the Li–Wu–Luo method (Li, et al. 1985). To reduce the number of repeated uses of a mutation in different pairwise comparisons, we took only the top one-third of the dS values, and computed the dN/dS ratios and their mean for the top one-third pairs. Fisher’s exact test of neutrality for sequence pairs was done in MEGA X platform to cross check dN/dS-based selection.

**Table 1:** The dN/dS ratios in the first wave of infection, the α variant, δ variants in Assam, and the omicron variant outside of Assam

Gene Name	Gene length (bp)	Average dN (SE)	Average dS (SE)	Average dN/dS
First wave				
ORF1ab	21,290	0.0715 (0.0142)	0.0684 (0.0133)	1.045
S	3822	0.1931 (0.0416)	0.2105 (0.0485)	0.917
ORF3a	828	0.0021 (0.00001)	0.0062 (0.00001)	0.339
E	228	0 (0)	0.0216 (0.00001)	0
M	669	0 (0)	0 (0)	*
ORF6	186	0.0079 (0.0001)	0 (0)	#
ORF7a	366	0.00009 (447E-05)	0.0169 (0.0004)	0.005
ORF7b	132	0 (0)	0.0414 (1.2985E-05)	0
ORF8	366	0.0013 (0.0001)	0.0159 (0.0002)	0.082
N	1260	0.0016 (5.28E-05)	0.0053 (8.3505E-05)	0.302
Average dN/dS ratio				0.448 (0.177)
Alpha variant				
ORF1ab	21,290	0.0002 (0.00004)	0.0025(0.0.0001)	0.080
S	3822	0.1681 (0.0949)	0.1646(0.0750)	1.021
ORF3a	828	0.0005 (0.0001)	0.0079(0.0002)	0.063
E	228	0 (0)	0(0)	*
M	669	0.0004 (0.0003)	0.0079(0.0001)	0.051
ORF6	186	0 (0)	0(0)	*
ORF7a	366	0 (0)	0(0)	*
ORF7b	132	0 (0)	0(0)	*
ORF8	366	0 (0)	0(0)	*
N	1260	0.0007 (0.0001)	0.0044(0.0001)	0.159
Average dN/dS ratio				0.277 (0.188)
Delta variant				
ORF1ab	21,290	0.0005 (5.9083E-06)	0.0012(9.7409E-06)	0.417
S	3822	0.1732 (0.0324)	0.2372(0.0374)	0.730
ORF3a	828	0.0004 (2.4662E-05)	0.0076(9.8678E-05)	0.053
E	228	0.0062 (5.3676E-05)	0(0)	#
M	669	0.0004 (6.3839E-05)	0.0082(5.3809E-05)	0.049
ORF6	186	0.0079 (6.2997E-05)	0(0)	#
ORF7a	366	0.0014 (0.0001)	0.0144(0.0001)	0.097
ORF7b	132	0 (0)	0.0448(0.0001)	0
ORF8	366	0.0005 (0.0001)	0.0149(2.6939E-05)	0.034
N	1260	0.0009 (1.96E-05)	0.0059(5.4534E-05)	0.153
Average dN/dS ratio				0.128 (0.050)
Omicron variant				
ORF1ab	21,290	0.4627 (0.0363)	0.5177(0.0417)	0.894
S	3822	0.2650 (0.0840)	0.2139(0.0595)	1.239
ORF3a	828	0.0008 (7.04E-05)	0.0084(0.0002)	0.095
E	228	0 (0)	0.0236(0.0005)	0
M	669	0.0030 (0.0001)	0.0112(0.0002)	0.268
ORF6	186	0 (0)	0(0)	*
ORF7a	366	0 (0)	0(0)	*
ORF7b	132	0.0022 (0.0004)	0.0402(0.0129)	0.055
ORF8	366	0 (0)	0(0)	*
N	1260	0.0014 (0.0001)	0.0053(0.0002)	0.264
Average dN/dS ratio				0.469 (0.197)

The cases with dS/SE < 1, where SE is the standard error, are excluded in the estimation of dN/dS and are indicated by #. *Indicates dS = dN = 0

The genome-wide average dN/dS ratio of the variants varied from 0.128 ± 0.050 to 0.469 ± 0.197 (Table 1). This result clearly reveals that overall, the VOCs of SARS-CoV-2 were under strong purifying selection as observed in previous studies [23]. However, some individual genes seem to be subject to positive selection. ORF1ab contains an overlapping open reading frame that encodes polyprotein (PP) PP1ab or PP1a depending on the a-1 ribosomal frameshift event. The PPs are cleaved to yield 16 non-structural proteins (nsp) [24]. The viral genes S, E, M, and N encode structural proteins, while ORF6, ORF7, and ORF8 participate in immune evasion [25–28]. ORF3a is a protein with ion-channel activity (viroporin) that activates the NLRP3 inflammasome [29]. Note that ORF1ab showed dN/dS > 1 (implying positive selection) in the FW and omicron variants. Among the structural genes, S showed dN/dS > 1 or close to 1 (positive selection) in all SARS-CoV-2 variants, while the other structural (E, M, and N) and the immune evasion (ORF6, ORF8, ORF7a, ORF7b) genes are under strong purifying selection in all the SARS-CoV-2 variants studied. ORF3a showed relaxed selection in fw compared to the strong negative selection in the α, δ, and omicron variants. The above found dN/dS-based selection on the genes were confirmed by a probability (p) value < 0.05 based on Fisher’s test of neutrality for sequence pairs.

### Differential expression of SARS-CoV-2 genes and its relation with Ct value

**Fig. 6:**
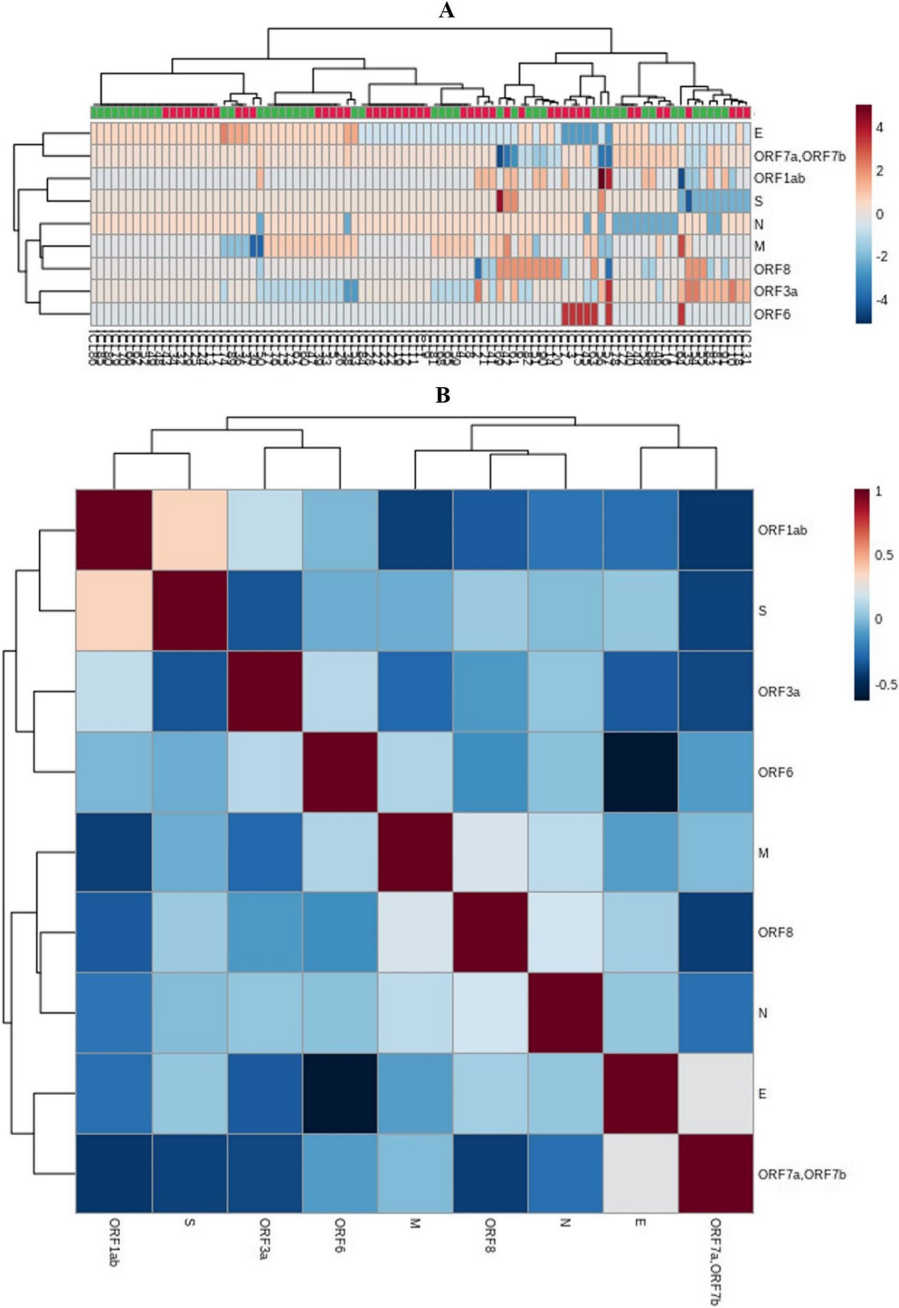
A heatmap of gene-expression levels (**A**) and a correlation coefficient matrix of the expression levels of genes of the studied 92 SARS-CoV-2 samples (**B**). **A** The heatmap represents the expression patterns of the genes of SARS-CoV-2 in different samples. High expression is shown in red and low expression in blue in terms of FPKM values (log2 scale) (see the colour bar at the right side). The entries on the right denote the gene names while those at the bottom denote the sample IDs. The hierarchical clustering of genes is based on their expression similarity in the 92 samples. **B** A correlation coefficient matrix of gene expression levels. The entries on the right and at the bottom denote the gene names and the colour bar on the right denotes Pearson’s correlation coefficient (r)

SARS-CoV-2 has a positive-strand RNA, and it expresses its genes by forming a negative sense antigenome, known as replication intermediate, which leads to formation of sub-genomic messenger (m) RNAs with capping and polyadenylation by a process known as discontinuous replication [30]. The RNAs extracted from the samples therefore carry the expressed mRNAs of SARS-CoV-2, which reflects their spatio-temporal quantitative expression in the host. Therefore, apart from using the sequencing reads for genome assembly, we also used the reads to estimate the expression level of SARS-CoV-2 genes. For this analysis, the reads were aligned against the SARS-CoV-2 reference genome (accession NC_045512) and subsequently processed for a reference-based assembly. The expression of a gene was quantified by “fragments per kilobase of exon per million mapped reads (FPKM)”. Additional file 8: Table S7 shows the expression levels of genes in the FPKM scale and Fig. 6a shows the heatmap of the expression of SARS-CoV-2 genes in different samples in normalized log2 scale. A gene is considered expressed if its FPKM is ≥ 1 in at least one of the samples. The hierarchical clustering (Fig. 6a) and correlation coefficient matrix (Fig. 6b) based on the expression levels of the genes classified the expressed genes into two clusters. Cluster 1 includes the genes for E and ORF7ab. Cluster 2 includes ORF1ab, S, N, M, ORF8, ORF3a, and ORF6, which can be further divided into three sub-clusters: i) ORF1ab and S, ii) N, M, and ORF8, and (iii) ORF3a and ORF6. Among them, the correlation coefficient of ORF6 transcripts (having average read depth and FPKM value of 359 ± 4.02 and 712 ± 5.69, respectively, Additional file 8: Table S7) with the PCR Ct (Ct refers to number of cycles after which the virus can be detected. A sample having higher virus load will show less Ct value then the sample with low virus load) value is -0.55 (p < 0.0001), which signifies that the expression of this immune evasion tentatively corresponds to the viral load in the Covid-19 positive human hosts however, this needs further validation from future studies. However, the other genes involved in immune evasion (ORF7a, ORF7b, ORF8) showed no correlation with the Ct value. Thus, among the immune evasion genes, only the ORF6 gene, which is subject to functional constraint, is negatively correlated with the Ct value of the patient. Therefore, a higher expression of the ORF6 gene implies a higher viral titre in the infected person.

## Discussion

Northeast India was a high burden area for Covid-19 both in the first and the second wave [31]. Therefore, we sequenced 92 samples of SARS-CoV-2 over the time frame of March to July, 2021 from Assam, India, to keep track of mutant variants. All of these 92 samples were found to belong to the δ variant clade, which is also seen in other parts of India over that time frame [32]. A comparison with the Pangolin database [7] revealed that 29.34% of our samples belonged to the B.1.617.2 lineage, 28.26% to the AY.33 lineage, 26.08% to the AY.16, 13.04% to the AY.4 lineage, and 1.08% to the AY.34 and the AY.37 lineages; the δ variant includes the lineages B.1.617.2 + AY*. Notably, we found 12 cases of AY.4 δ variant, indicating that this region carries the SARS-CoV-2 AY.4.2 lineages of δ variants, which are suspected to cause severe illness or deaths in India [33]. The genome-wide amino acid variant analysis revealed that a group of 13 variants transmitted together with a high frequency in Assam. This suggests that these variants were inherited together and represented a haplotype of the δ variant. So far, the spread of SARS-CoV-2 in different regions of the world has been tracked as VOC [1] and except for a few studies, attention has not been given to the spread of haplotypes or sub-haplotypes in a region [11, 34]. A previous study showed that there were clusters of sub-lineages of the δ variant across different regions of Germany and the United Kingdom [35]. Therefore, we hypothesize that a specific haplotype of the δ variant highly transmitted in Assam. The set of variants of the haplotype changed in two ways. First, there was a selective sweep of 13 pre- existing haplotype variants, including 4 variants (2 in S protein and 2 in ORF8), which have a frequency <91% outside Assam in the same lineage and over the same time frame. The increase in frequency in Assam of the 4 variants might be due to selective advantage in this region, or else some of them are carried as hitchhiker because of their linkage with advantageous variants in S protein [36]. As noted above, the selective advantage might be instead owing to the variant on ORF8, which is tightly linked to the S-protein locus. However, this needs further validation in future studies with more extensive data. Second, 10 variants of the haplotypes were reduced in frequency in Assam, likely by mutation or recombination perhaps due to their weak linkage with the S-protein (and the ORF8 locus). Recombination is widespread in coronavirus due to switching RNA-synthesizing genes from one template to another [37, 38]. Many of the previous studies have shown that the successive evolution of SARS-CoV-2 variants involved repeated episodes of recombination [20, 21, 39]. In India, especially in the second wave of infection, although the reported cases of infection from different states were mostly the δ variant, the rate of transmission and pathogenicity significantly differed among the states of the country. The actual reason for this phenomenon remains unclear, perhaps due to little understanding of haplotype transmissibility in different regions.

We observed frequent changes of amino acids to stop codon or frame-shift mutations in the SARS-CoV-2 genome of Assam. This is in accordance with the previous reports that SARS-CoV-2 may express truncated proteins or frame-shifted proteins and these events increase the quasispecies dynamics of SARS-CoV2, which might provide SARS-CoV-2 genetic fitness on cell tropism and host range [16, 40].

We calculated the dN and dS values for each of the genes of SARS-CoV-2 VOCs that evolved in different timelines. The calculated dN and the dS values are smaller compared to those observed in other RNA viruses [41]. The low dS values may lead to overestimation of dN/dS. To minimize this chance, we only used the top one-third of the dS values with the additional condition of dS/SE < 1. Our average dN/dS values of SARS-CoV- 2 VOCs are not strikingly different from those in the previous studies [42–44], implying that overall the SARS-CoV-2 genome is evolving under strong purifying selection. The calculated gene-wise dN/dS for FW, α, δ and omicron revealed that the gene for ORF1ab was under positive selection in the FW and omicron variants while S-protein was under positive selection in all the SARS-CoV-2 variants studied. ORF3a may be under relaxed negative selection in FW compared to other genes. Thus, the S-protein might have undergone positive selection in some of the VOCs [45]. ORF1ab and ORF3a have been reported to undergo positive selection that drove the early evolution of SARS-CoV-2 [44]. However, for ORF1ab, this was true only for the α and omicron variants. The structural genes, except S, and the immune evasion genes in SARS-CoV-2 were under strong purifying selection.

The expression pattern of the genes of SARS-CoV-2 relates to their characteristicpattern of evolution. Most importantly, we found that ORF6, which revealed high evolutionary conservation, showed differential expression levels among different samples collected from infected persons. Moreover, ORF6 showed a negative correlation with the Ct value of samples. This data tentatively indicates a positive correlation between upregulation of ORF6 and an increase in virus titre in a host. The cytokine profile and inflammatory response are different in the case of SARS-CoV-2 infection [46]. Previous studies have shown that ORF6 regulates immune escape in the human host by inhibiting STAT1nuclear translocation to overcome the interferon mediated antiviral response and also by binding with Nup98-Rae1 complex thereby inhibiting the nuclear import pathway [26]. Therefore, we may assume that upregulation of ORF6 is an essential determinant for the successful invasion of SARS-COV-2 in a human host, for this reason, this gene shows extraordinary functional conservation in evolution. However, this needs further validation from the future studies.

## Conclusion

This study is a detailed analysis on the SARS-CoV-2 genome, to understand its dynamic evolution from a regional perspective. Here we have found that a haplotype of delta variant underwent complete selective sweep in a population, we evokes the need to focus on haplotypes of SARS-CoV-2 variants for effective management of viral pandemic regionally in future.

## Materials and methods

### Ethical clearance

The Institute of Advanced Study in Science and Technology (IASST) has a Covid-19 testing laboratory and research facility (BSL-2 approved laboratory) under Indian Council of Medical Research (ICMR). The research work under this study entitled “Surveillance of SARS-CoV-2 variants of concern in Assam by whole genome sequencing” was approved by the institutional ethnical committee (IEC(HS)/IASST/1082/2021/6). The survey data and consent forms were collected from participating individuals by following the standard ethical guidelines.

### Collection of samples and metadata

The nasopharyngeal and throat swabs were collected as per the ICMR protocol in viral transport media from Covid-19 positive patients (Ct value <30) showing symptoms including respiratory problems, fever, cough and cold, sneezing. Post vaccination patients were also included in this study. A total of 92 samples were collected from different districts of Assam including Kokrajhar, Bongaigaon, Goalpara, Kamrup Metro, Nagaon, Darrang, Golaghat, Tinsukia and Morigaon and processed for whole genome sequencing. The sampling details are given in Additional file 9: Table S8 and also shown in Additional file 11: Fig. S2. While one sample (ICL5) was collected on 6th march, 2021, the other 91 samples are collected from 27 May, 2021 to 24 July, 2021.Extraction of viral RNA and SARS-CoV-2 detection assays RNAs were extracted from nasopharyngeal swab and oropharyngeal swab samples [47] using Qiagen QIAmp® Viral RNA Mini Kit (250) following manufacturers protocol. The RNA was extracted from SARS-CoV2 samples and eluted in 50 μl elution buffer. 5 μl of RNA was used in each SARS-CoV-2 detection assay. Real-time PCR assay was performed using the CoviPath^TM^ COVID-19 multiplex kit (Thermo Fisher, Cat no. A50780) using Agilent AriaMx real-time PCR system with the following thermal condition: 2 min at 25°C for UNG incubation, 10 min at 53°C for reverse transcription and 2 min at 95 °C for activation of Taq polymerase followed by 40 cycles of 3 sec at 95 °C and 30 sec at 60 °C. The relative abundance was calculated using the Ct method [48]. Abundance levels of the N gene and ORF genes were normalized to that of RnaseP and presented as the Ct value, which was inversely correlated to the gene expression level. These values from the multiplex PCR system were compared with the gene expression profile generated using the sequencing reads as mentioned in the section “Assessment of the expression level of genes in SARS-CoV2’ below.

### Sequencing of SARS-CoV2 genome

Whole-genome sequencing of the viral isolates were performed in the Illumina Hi-SeqX platform using QIAseq DIRECT SARS CoV-2 kit (Qiagen, Cat no. 333898). For this purpose, sheared SARS-CoV2 genome was amplified with specific primer and the amplicons were used for library preparation and sequenced in HiSeqX to generate 2x150bp reads with >90% reads above Q30 value. The sequencing of RNA samples was performed by MedGenome Labs Ltd., Bangalore, India.

### Data processing

The raw fastq files were checked for quality and quantity using FastQC (v. 0.11.9) tool [49]. During filtering, reads were considered for further processing having Phred quality score (Q30) > 80%, GC content <35% & >45. Adapters and the contamination were removed from the fastq file using cutadapt (v.2.9 tool) [50]. The raw reads were aligned against the host reference genome (the human genome). The unaligned pair-end reads were aligned to the SARS-CoV-2 reference genome downloaded from NCBI RefSeq (NC_045512.2). Alignment was performed using BWA aligner (v.0.7.12) [51]. Reads having alignment score <80% were discarded during the analysis. Genome length, read depth, mapping statistics, total reads, GC%, total data generated were provided as Additional file 2: Table S2. Consensus sequences generated for each sampl were used for downstream analysis.

### Quality control of raw reads and subsequent genome mapping

We obtained 92 wgs of SARS-CoV-2 from Assam for a genome-wide comparative study. The mapping and alignment statistics of the reads of the sequences are shown in Additional file 10: Table S9. On average, more than 6 million pair-end reads were generated for each sample. Around 90% of raw reads passed the quality filter (Q30%) and 95% of the filtered reads mapped to the reference genome of SARS-CoV-2 from Wuhan, China (accession NC_045512). The mapping statistics of the reads are comparable to those in previous studies [52]. The mapped ‘binary alignment files’ were subsequently processed for reference-assisted assembly, which yielded a single contig in each case with average length of 29 Kb. The raw reads and the final wgs of the 92 samples were submitted to the GISAID database (see accession numbers in Additional file 2: Table S2).

### Variant calling and annotation

GATK variant caller (V4.1.0.013) [17] was used for variant detections. Low quality variants were filtered based on read depth and allele frequency. The aligned reads and the reference fasta file of Wuhan strain (accession no. NC_045512.2) were sorted prior to variant calling using Samtools [53]. We used the Picard tool for file conversion and the output BAM files were sorted by coordinates. The read groups were added using “Add or Replace Read Groups” functions to avoid generating error regarding header/groups. Duplicate reads were identified and removed through ‘Mark Duplicates’ function with ‘remove_duplicates’ as true argument. Subsequently, we performed local realignment around indels to reduce the mapping error of the genomic region that contains indels. Mate-pair information was verified by ‘Fix Mate Information’ function on Picard. Base quality recalibration was performed for each sample to remove any systematic bias during the variant discovery process. Subsequently, we used ‘Haplotype Caller’ function with phred-scaled confidence threshold of 20 for the detection of SNPs and indels. The identified variants in variant calling format (VCF) output file were annotated and the effects of the genetic variants using SnpEff 4.5covid19 tool [54]. The variant class, amino acid changes and other relevant annotations were added to the variants. Variant positions, quality, read depth, allele frequency, zygosity, annotations and impact of annotations were provided in Additional file 6: Table S5. The variants of the SARS-CoV-2 found in our study were compared with GISAID database to find the novel and pre-existing variants vis-à-vis the frequency of pre-existing variants globally in comparison to Assam.

### Phylogenetic analysis and lineage study

For the phylogenetic reconstruction, we used the sequences derived from Assam along with database sequences representing major VOCs (Additional file 4: Table S3). A total of 184 sequences were aligned using the Fourier transform algorithm (MAFFT) [55]. We used the progressive alignment strategy and the gap opening penalty and gap extend penalty was set to 1.53 and 0.123, respectively. The evolutionary history was inferred by using the Maximum Likelihood method and the Tamura-Nei model [56]. The sequences were aligned for a total length of 29,903 bp. The bootstrap consensus tree inferred from 1000 replicates [57] was taken to represent the evolutionary history of the taxa analysed. Branches corresponding to partitions reproduced in less than 50% bootstrap replicates are collapsed. Initial trees for the heuristic search were obtained automatically by applying the Neighbor-Join and BioNJ algorithms to a matrix of pairwise distances estimated using the Tamura-Nei model, and then selecting the topology with superior log likelihood value. Evolutionary analyses were conducted in MEGA11 [58]. The phylogenetic tree was refined using Randomized Axelerated Maximum Likelihood (RaxML) [59] with a model type Nucleotide and substitution GTR+gamma model (-m GTRGAMMA). iTOL programme [60] was used for the visual representation of the tree. Lineages were assigned to sequences using the Pangolin tool (version v3.1.14, pangoLEARN version 28-09-2021) [7]. Sequences with lineage and clade information were provided in Additional file 5: Table S4.

### Assessment of selection on genes of SARS-CoV-2

We collected the genomes of SARS-CoV-2 prevalent in FW, i.e., from January 2020 to June 2020, and those that appeared from November 2020 to February 2021 in UK, designated as the α variant, and also those that appeared from February 2021 to June 2021 in India, designated as the δ variant. The dataset altogether constituted 184 genomes (Additional file 1: Table S1). We categorized the dataset as FW, α and δ because we are interested to see the selection on the genomes within the categories that appeared in different timelines that mostly infected specific populations. The sequences were subjected to codon-based alignment using MAFFT as explained above. The dN and dS values within each of the categories were computed by the Li–Wu–Luo method, using MEGA6.0.

### Assessment of the expression level of genes in SARS-CoV2

To identify the gene expression pattern across samples from Assam, we used cufflink [61]. To significantly improve the accuracy of transcript abundance estimates, we used fragment bias correction using reference fasta file (NC_045512.2). The datasets were normalized using the classic-fpkm library normalization method. Average length of the fragment and fragment length standard deviation were set at 200 bp and 80 bp, respectively. We filtered out transcripts with very low abundance. The other parameter was set as —max-intron-length none, binomial test used for false positive spliced alignment filtration (–junc-alpha 0.001), –small-anchor-fraction 0.09, –overhang-tolerance 8, –min- intron-length 50bp, –trim-3-avgcov-thresh 10. Maximum likelihood estimation of abundances for iterations was set to default value of 5000. Samtools was used to sort the BAM files. The Wuhan reference genome annotation file (GCF_009858895.2_ASM985889v3_genomic.gtf) was used to quantify the gene abundances for each sample. The Fragments Per Kilobase of transcript per Million mapped reads (FPKM) values were used for further analysis. Abundance values of ORF1ab, S, ORF3a, E, M, ORF6, ORF7a, ORF7b, ORF8, N, ORF10 genes along with the samples were provided in Additional file 8: Table S7.

### Supplementary Information

**Additional file 1:** Phylogenetic assignment of derived sequences based on PANGOLIN database.

**Additional file 2:** Derived and database sequences of SARS-CoV-2 used in this study, together with strain information and WHO nomenclature.

**Additional file 3:** Genome-wide variation map.

**Additional file 4:** Nucleotide variants detected on the derived sequences in comparison to Wuhan strain.

**Additional file 5:** Overall nuclotide and amino acid variation found in the derived sequences.

**Additional file 6:** Gene-wise amino acid variation found in the derived sequences.

**Additional file 7:** Fequency of observed amino acid variants on the genome sequences of SARS-CoV-2 on the GK clade outside Assam (timeline: March - July, 2021).

**Additional file 8:** Expression level of SARS-CoV-2 genes in FPKM scale.

**Additional file 9:** Sampling details.

**Additional file 10:** Mapping and alignment statistics of the derived sequences of SARS-CoV-2.

**Additional file 11:** Map showing location of sampling.

## Data Availability

The sequences generated in this study are submitted to GISAID database and can be accessed using the accession number given in Additional file 2: Table S2.
