# Supplementary figures and images for: Genome analysis of SARS-CoV-2 isolates from a population reveals the rapid selective sweep of a haplotype carrying many pre-existing and new mutations
Source: Virol J. 2023 Sep 1;20:201. doi: 10.1186/s12985-023-02139-3 (PMC10474745; doi:10.1186/s12985-023-02139-3)

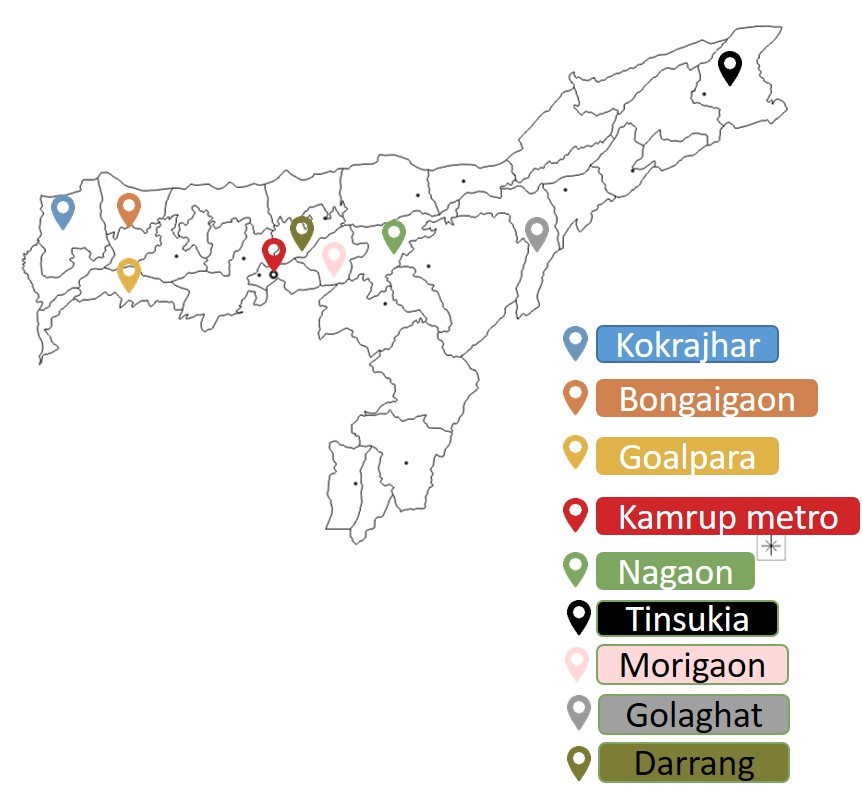

Supplement: Supplementary file 2 — Additional file 2: Derived and database sequences of SARS-CoV-2 used in this study, together with strain information and WHO nomenclature. [file 12985_2023_2139_MOESM2_ESM.jpg]

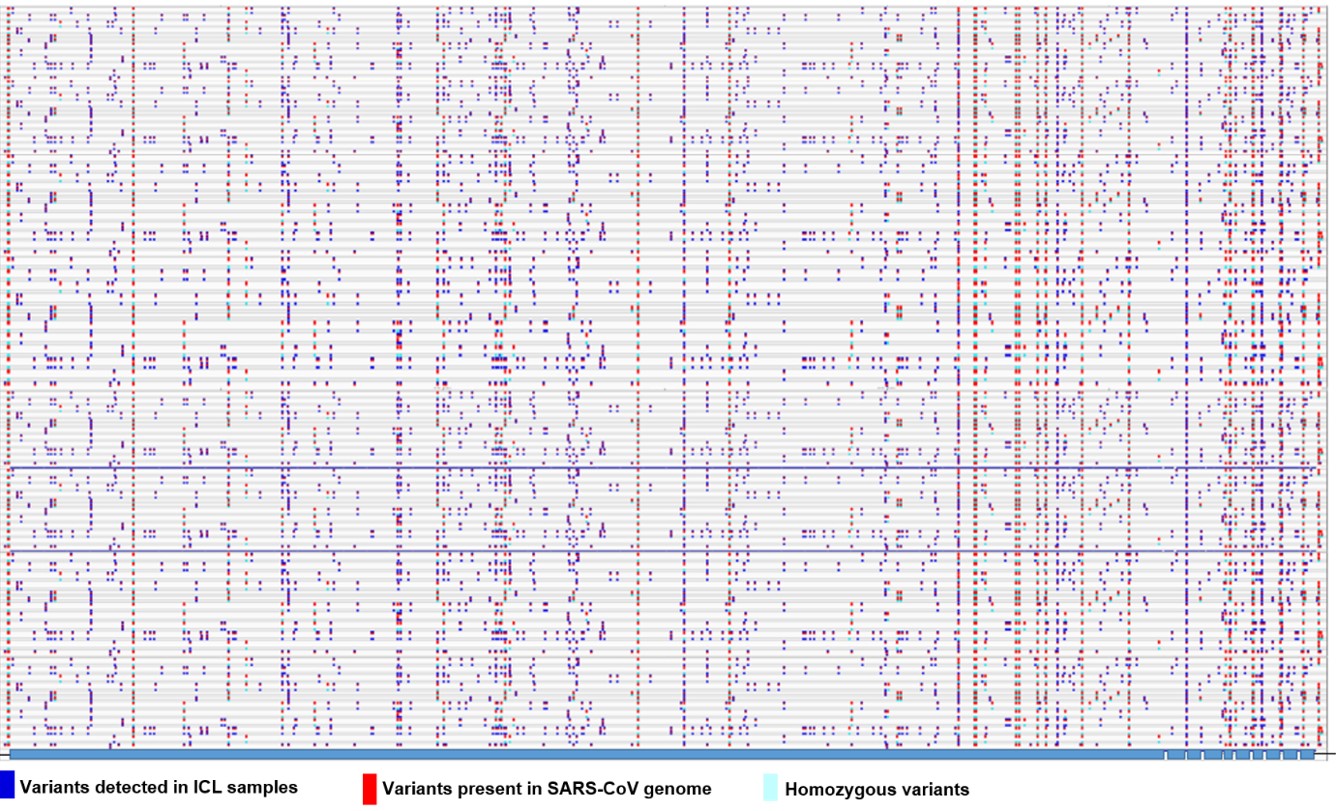

Supplement: Supplementary file 5 — Additional file 5: Overall nuclotide and amino acid variation found in the derived sequences. [file 12985_2023_2139_MOESM5_ESM.jpg]
